# Exercise-induced local sweating: Greater reduction in women than men with sunscreen use

**DOI:** 10.1080/23328940.2024.2396198

**Published:** 2024-09-10

**Authors:** Julián C. Garzón-Mosquera, Luis F. Aragón-Vargas

**Affiliations:** a Doctoral Program in Science, University of Costa Rica (UCR), San José, Costa Rica; b Physical Education and Sport School, University Professor, University of Costa Rica (UCR), San José, Costa Rica; c Physical Education and Sport School, Human Movement Science Research Center (CIMOHU-UCR), San José, Costa Rica

**Keywords:** Thermoregulation, sweating, sunscreen, skin, heat

## Abstract

Sunscreens may affect thermoregulation and sweating during exercise in warm climates. In this study, we compared the effects of three sunscreens on local sweating rate (LSR) during exercise under controlled conditions (32°C, relative humidity 55%). Fifteen subjects (8 men, 7 women) underwent 20-min cycles in four randomized conditions: control (CON), sunscreen A (SSA), sunscreen B (SSB) and sunscreen C (SSC). LSR was measured by a patch on the scapular region (*p* < .001). CON showed higher LSR (182.21 μL/min·dm^2^, CI95% 168 to 195 μL/min·dm^2^) compared to SSA (142.10 μL/min·dm^2^, CI95% 128 to 155 μL/min·dm^2^), SSB (158.06 μL/min·dm^2^, CI95% 144 to 171 μL/min·dm^2^), and SSC (159.00 μL/min·dm^2^, CI95% 145 to 172 μL/min·dm^2^). In men, SSA showed lower LSR compared to CON, SSB, and SSC (*p* < .05). On the other hand, no statistically significant differences were found in LSR between SSB, SSC, and CON conditions. In women, CON was superior to all sunscreens in LSR (*p* < .001), and there was no difference between them (SSA, SSB, SSC, *p* > .05). Sunscreen reduced LSR during moderate exercise in a hot and moderate humidity environment compared to CON, especially SSA in men and all sunscreens in women.

## Introduction

Controlling body temperature through sweat evaporation during exercise in the heat is essential to maintain homeostasis and prevent risks like hyperthermia and cardiovascular issues [1]. Exercise in warm conditions increases body heat production, requiring efficient dissipation through sweating to avoid dangerous temperature rises and ensure thermal homeostasis [2]. Sweating, a key thermoregulation mechanism, reduces internal temperature as sweat evaporates, releasing water and electrolytes [3]. This process facilitates heat exchange between the body and the environment, lowering skin and subcutaneous blood vessel temperatures [4,5]. External factors like high ambient temperature, humidity, and solar radiation heighten the risk of overheating during physical activity [2]. Prolonged exposure to ultraviolet (UV) rays can cause burns and heat exhaustion, especially when exercising outdoors [6]. Therefore, sun protection measures like wearing appropriate clothing and applying sunscreen are crucial to prevent skin damage and maintain thermoregulation [7,8]. However, sunscreen applied directly to sun-exposed skin may interfere with effective sweating.

Beyond the importance of sunscreen use for outdoor physical activity, the specific type of sunscreen (organic, inorganic, and natural chemical derivatives [9]) is a relevant aspect to consider. In this regard, Aburto-Corona & Aragon-Vargas [10] have evidenced that some inorganic sunscreens may have antiperspirant properties, affecting sweating; specifically, they found that the application of a particular inorganic sunscreen significantly (*p* < .01) reduced the rate of local (scapular) sweating during exercise in hot (30°C) and humid (58%) conditions (99.3 μL/min·dm^2^), compared to the application of a specific organic sunscreen (114.8 μL/min·dm^2^). These findings indicate that the type of sunscreen used, especially specific ingredients in it, could influence LSR.

Wells et al. [11] demonstrated that sunscreen application during exercise in hot, low humidity conditions (35.2°C, 15.3 mmHg water vapor pressure, ~27% relative humidity) significantly increased skin temperature from the 12th minute onwards compared to no sunscreen use, while core temperature and physiological strain (heart rate, VO2, weight loss) were unaffected. The elevated skin temperature suggests sunscreen hindered sweat evaporation and heat dissipation from the skin. Notably, the skin temperature response with sunscreen resembled that of high humidity trials initially until sweat saturation occurred earlier without sunscreen. The core-to-skin temperature gradient was also significantly reduced by sunscreen application in the low humidity condition after 12 min of exercise, further reflecting impaired dry heat loss. These findings suggest sunscreen can compromise thermoregulation during exercise in dry heat by disrupting evaporative and dry heat dissipation mechanisms.

These results are congruent with the subsequent study done by House & Breed [12], who also found that sunscreen application in warm, dry environments (40°C − 20% relative humidity) decreases sweat evaporation, which negatively affects thermoregulatory capacity during exercise. It should be clarified that the study indicated lower sweat evaporation rather than lower sweat production with the use of sunscreen [12]. Both investigations suggest that under certain unfavorable environmental circumstances, some sunscreens may interfere with heat dissipation in the skin through sweat evaporation, leading to increased heat stress.

Some concerns arise around using sunscreens during exercise in the heat due to potential impacts on thermoregulatory function. Certain sunscreen ingredients like dimethicone [13,14], C10–30 alkyl acrylates [15], glyceryl stearates [16] and hydrogenated coconut glycerides [17] may have occlusive properties that obstruct sweat gland function and evaporative cooling. Though UV filters absorb radiation, these other components could inadvertently interfere with local sweat production during exercise. Investigating the potential occlusive effects of sunscreens on sweating and thermoregulation is therefore crucial.

While prior studies have presented contrasting findings on the impact of sunscreens on sweating rates, this highlights a critical gap in our understanding that warrants further rigorous investigation. Ou-Yang et al. [18], Connolly & Wilcox [19] and Fisher et al. [20] reported no significant effects, whereas Aburto-Corona & Aragon-Vargas [10] observed differences in local sweat rates (LSR) with specific sunscreen formulations. These discrepancies underscore the need for comprehensive research to elucidate the potential thermal and thermoregulatory influences of various sunscreen compositions during exercise in the heat. Furthermore, the discrepancies between the studies may be attributed to differences in the specific sunscreen formulations used in the experiments, which further underscores the need for extensive research in this area.

In addition, other studies such as Wells et al. [11] and House & Breed [12] point out negative effects of sunscreen on effective sweat evaporation. Considering the controversies in previous studies, this study hypothesizes that certain types of sunscreens, particularly those with occlusive ingredients, may negatively affect the local sweating rate (LSR) during physical activity in warm and moderately humid conditions, thereby compromising thermoregulation. To test this hypothesis, the study aims to compare the effects of three water- or sweat-resistant sunscreens with sun protection factor (SPF) ≥50 on LSR during 20 minutes of physical activity in these conditions. The results will provide new evidence on how the chemical agents used as UV filters (UVA and UVB), and secondary components in these sunscreens impact sweating under heat stress conditions. If a negative effect on LSR is confirmed, it would warrant further research on the broader implications of sunscreen use for whole-body sweat rates, thermoregulation, and physical performance in the heat.

## Methodology

**Figure 1.**
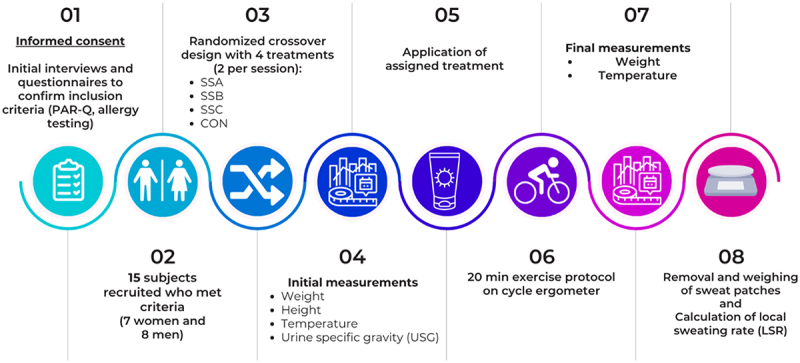
Diagram of methodology to evaluate impact of sunscreens on local sweating rate. PAR-Q, the Physical Activity Readiness Questionnaire for Everyone; SSA, organic chemical sunscreen Nivea®; SSB, inorganic chemical sunscreen Cetaphil®; SSC, inorganic chemical sunscreen Repaskin®.

The protocol was approved by the University of Costa Rica Institutional Review Board, CEC-524-2022 (**Figure 1**). All participants provided informed consent prior to any procedures. After conducting unstructured interviews to confirm that the potential subjects had no endocrine, renal or cardiac problems, that they were not taking any medications that could alter sweating, and did not have a history of heat illness, and that the women were not pregnant, the Physical Activity Readiness Questionnaire for Everyone (PAR-Q) [21] was applied and an individual allergy test was performed with the sunscreens to be used (appendix Table A1), wherein a small amount of each sunscreen formulation was applied on the inner forearm, and the area was observed for any potential skin reactions over a short period (10 min) prior to proceeding with application on the scapular areas. Each participant was asked not to engage in strenuous physical activity 24 h before each session. Fifteen physically active subjects met the criteria: 7 females with age = 22.8 ± 4.0 years (mean ± S.D.), height = 157.5 ± 4.0 cm, body weight = 57.2 ± 6.0 kg, and 8 males aged = 26.2 ± 5.1 years, height = 174.3 ± 7.4 cm, and body weight = 72.4 ± 10.5 kg).

## Procedure

The evaluation method has been validated and previously published [10,22]. Briefly, using a repeated-measures, crossover-randomized design, four conditions (tested in pairs) were evaluated in all subjects: sunscreen A (SSA; Nivea® sun SPF 50, Beiersdorf, Hamburg, Germany), sunscreen B (SSB; Cetaphil® sun SPF 50+, Galderma, Fort Worth, Texas, United States), sunscreen C (SSC; Repaskin® sunscreen photoprotector SPF 50+, Valencia, Spain), and control (CON; no sunscreen). The sunscreens used meet the criteria of having an SPF of 50 or higher and being water and sweat resistant (**Appendix Table A1**).

All subjects were instructed to avoid taking diuretics for 24 h before testing, and to drink at least 1 liter of water the night before. To ensure unrestricted access to the scapular area, women were specifically asked to wear sports tops that left each scapula completely uncovered, ensuring the area remained exposed throughout the testing period. Men were asked to go shirtless to achieve the same result. The scapula was not covered in women at any point during the tests, as the design of their sports tops allowed full exposure of the scapular region.

Once the conditions were assigned, baseline urine-specific gravity (USG) measurements were performed using a refractometer (ATAGO®, model URC-Ne, d 1,000-1,050, Minato-ku, Tokyo, Japan) with a reference for euhydration USG ≤ 1.020, along with initial nude, dry body weight (e-Accura®, model DSB291, Qingpu, Shanghai, China), height (SECA®, model 286, Hamburg, Germany) measurements in a private area to ensure privacy. Tympanic temperature (Tt) was recorded pre- and post-exercise as an approximation of body temperature of each participant, for descriptive purposes only (ThermoScan Pro 4000; Braun GmbH, Kronberg, Germany).

For the application of each condition, the left and right scapular region [23] were used following the methodology described by Aburto-Corona & Aragón-Vargas [10,22] to test 2 conditions per subject in each session. Conditions were randomly assigned to the left or right scapular region (bounded area) and to the first or second day of testing. The scapular area is hairless, has high sweat rates, and, notably, sweating rates are similar between contralateral sides [23,24].

The assigned condition was applied over the area of the sweat patches (16.17 cm^2^) at a dose of 2 mg/cm^2^ [25], which amounted to 32.34 mg of sunscreen applied over the entire area with the help of a stencil. Using a syringe, the calculated 32.34 mg amount was distributed evenly over the area by paddle, 20 min before starting the exercise protocol [10,22], the skin was cleaned with a damp alcohol wipe before application, leaving it dry and clean, and the researchers wore gloves during the process; any excess sunscreen was carefully removed, following the methods described by Aburto-Corona & Aragón-Vargas [10,22]. Sweat patches with an area of 4.9 × 3.3 cm = 16.17 cm^2^ (MSX − 6446, 3 M Medical Sciences, Brookings, S.D., United States) were then firmly adhered to the treated scapular area, creating an isolated and securely sealed collection surface. This watertight adhesive seal prevented evaporation and lateral sweat migration, ensuring only sweat produced within the bounded patch area was collected.

Each patch was identified for the condition to be used on each subject. Prior to placement on the skin, the patches were individually weighed using a 1 mg precision scale (model GX200; A&D Company, Limited, Tokyo, Japan), recording their weight and the condition for which it was used on each subject. Patches were placed on the demarcated scapular area prior to exercise. After the initial weighing, the patch was only removed from the airtight bag immediately before being applied to the subject’s skin.

The environmental conditions within the climate-controlled room at each session were 32°C and 55% relative humidity, monitored with a heat stress device (Questemp36® 3 M, Oconomowoc, WI, USA). The exercise protocol was performed on a stationary bike (Schwinn AC Performance Plus, Vancouver, WA, USA) for 20 min, pedaling at an intensity between 65% and 75% of each subject’s maximum heart rate, monitored with a Polar® heart rate monitor (Model FT7, Kempele, Finland). Maximum heart rate (MHR) was calculated using the formula of Tanaka et al. [26]: 208 - (0.7·age). Additionally, subjects were instructed to maintain a subjective perception of exertion between 12 and 15 points on a 20-point scale [27]. No food or beverages were consumed by the subjects during the exercise protocol.

At the end of the sweat collection period, the patches were promptly removed from each subject, placed back into the same airtight, hermetic bags they originated from, and then weighed to record the post-exercise weight of each patch. Additionally, the post-exercise body weight (BW) of each subject was recorded, together with Tt. The local sweating rate (LSR) was calculated according to Maughan et al. [28], Smith & Havenith [24], Morris et al. [29], and Aburto-Corona & Aragón-Vargas [10,22]. An equivalence was considered where 1 g of sweat equals 1 ml. The volume of sweat obtained was divided by the duration of the test (20 min) and the area covered by each patch (0.1617 dm^2^). In this way, the LSR was calculated in μL/min·dm^2^.

## Statistical analysis

The descriptive statistics of the subjects are presented: age, height, initial and final BW, environmental temperature, relative humidity, tympanic temperature, body surface area [30]. Normality and heteroscedasticity of the data, as well as equality between conditions, were verified for the following variables: initial urine-specific gravity, initial BW, average heart rate, environmental temperature and relative humidity, and initial Tt. Verification was performed by analysis of variance. A comparative analysis between conditions (paired two-sample t-test) was applied for initial and final body weight.

The main dependent variable (LSR) was analyzed using a least-squares test with a two-way interaction (sex * condition) and subjects as a random effect within the model, together with a least-squares contrast of means when significant differences were detected. Analyses were performed with RStudio 2023.06.0 + 421 (graphics) and JMP® Pro 17 (SAS Institute, Inc., Cary, NC, USA).

## Results

Descriptive data are presented as a mean ± standard deviation (SD). Males presented greater age, height (cm), body surface area (dm^2^) and initial body weight (kg) compared to females, these differences being significant (26.3 ± 5.2 years and 22.9 ± 4.0 years; *p* = .006), (174.36 ± 7.47 cm and 157.58 ± 4.03 cm; *p* < .001), (186.89 ± 2.39 dm^2^ and 158.17 ± 2.55 dm^2^; *p* < .001) and (72.43 ± 10.58 kg and 57.28 ± 6.06 kg; *p* < .001). A post-hoc statistical power analysis was performed to confirm that a sample of 15 participants was adequate for power >0.85, with α < 0.05 and a specific, meaningful mean difference (40.11 μL/min·dm^2^). Additionally, a detailed analysis for the primary comparison between males and females indicated that, with 7 women and 8 men, the study achieves a power of 0.90. This analysis used a combined standard deviation of 12.86 μL/min·dm^2^ and validated the sample size as sufficient to detect significant differences in local sweating rate between genders, ensuring robust statistical reliability for our gender-based comparisons.

The variables described in Table 1, were included as covariates in a mixed model with local sweating rate as the dependent variable, and condition and sex as fixed factors (Table 1). It was evident that none of these covariates significantly influenced the dependent variable (*p* > .05), and there were no significant differences in basal, environmental or exercise conditions between conditions (*p* > .05).

**Table 1.** Baseline values and covariance analysis.

	Condition mean (CI 95%)				Analysis of covariance	
Variables	SSA	SSB	SSC	CON	*F* value	*p* value
Body surface area (dm^2^)	173.3 (162.1 to 184.6)	173.4 (162.2 to 184.6)	173.5 (162.3 to 184.7)	173.6 (162.4 to 184.4)	3.803	.057
Mean heart rate (bpm)	134.0 (131.4 to 136.6)	134.2 (131.8 to 136.5)	134.9 (131.8 to 138.0)	134.0 (131.1 to 137.0)	0.067	.797
USG	1.014 (1.012 to 1.015)	1.014 (1.012 to 1.015)	1.014 (1.013 to 1.015)	1.014 (1.012 to 1.015)	0.289	.594
Initial body weight (kg)	65.28 (58.69 to 71.88)	65.30 (58.74 to 71.86)	65.39 (58.83 to 71.94)	65.46 (58.87 to 72.04)	3.159	.082
Body weight change (kg)	0.26 (0.14 to 0.38)	0.30 (0.23 to 0.38)	0.28 (0.21 to 0.35)	0.27 (0.21 to 0.34)	2.933	.094
Pre-exercise Tt (°C)	36.8 (36.5 to 36.8)	36.7 (36.4 to 37.0)	36.7 (36.5 to 36.9)	36.7 (36.5 to 36.8)	2.603	.114
Environmental temperature (°C)	35.1 (35.0 to 35.1)	35.2 (35.1 to 35.3)	35.1 (35.0 to 35.3)	35.1 (35.0 to 35.2)	0.621	.435
Relative humidity (%)	55.1 (55.0 to 55.2)	55.1 (55.0 to 55.3)	55.1 (55.0 to 55.3)	55.2 (55.1 to 55.3)	2.284	.138

USG, urine specific gravity; CI, confidence interval; dm2, square decimeters; bpm, beats per minute; °C, degrees Celsius; Kg, kilograms; Tt, Tympanic temperature; *CON, control; SSA, sunscreen a; SSB, sunscreen b; SSC, sunscreen c*.

**Figure 2.**
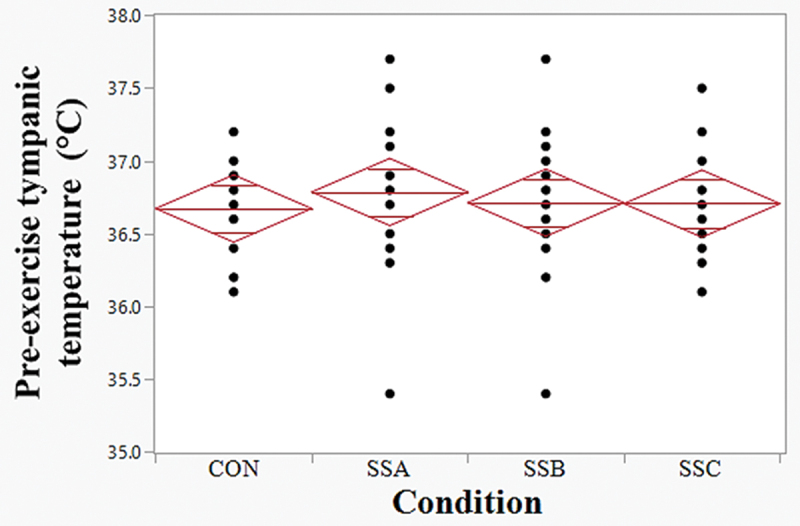
Pre-exercise tympanic temperature (mean, IC 95% and individual values). CON, control; SSA, sunscreen a; SSB, sunscreen b; SSC, sunscreen c; The diamond of the mean encompasses the 95% confidence interval for the mean of each condition, with a horizontal line at the mean in the middle of the diamond (blue lines).

Pre-exercise Tt (**Figure 2**) and BW did not show significant differences between conditions (*p* > .05). For each condition, the total sweating rate (estimated from the recorded body weight change) during exercise time (20 min) did not show significant differences among conditions (*p* = .873): CON (M ± SD, 278.0 ± 118.4 mL/20 min), SSA (264.0 ± 214.4 mL/20 min), SSB (309.3 ± 128.8 mL/20 min), SSC (286.7 ± 128.1 mL/20 min).

**Figure 3.**
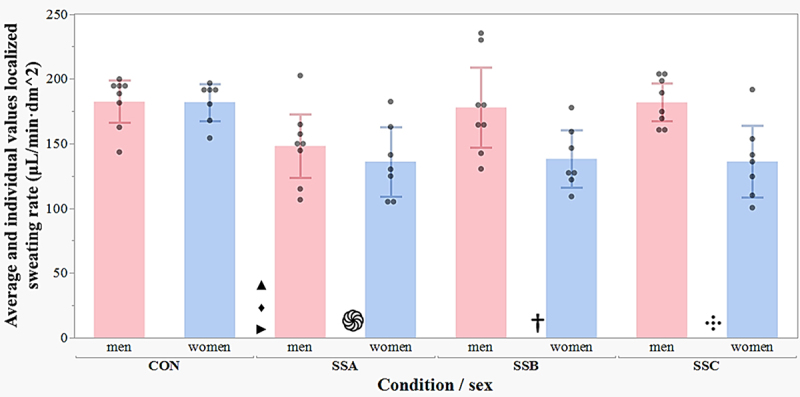
Local sweating rate between conditions and sex (mean, individual values with 95% confidence intervals). CON, control; SSA, sunscreen A; SSB, sunscreen B; SSC, sunscreen C; •, individual value; the significance symbol is at the left side of the corresponding bar; ▴, *p* < .05 men (CON to SSA); ♦, *p* < .05 men (SSA to SSC), ▸, *p* < .05 men (SSA to SSB); ֍, *p* < .05 women (CON to SSA); †, *p* < .05 women (CON to SSB); 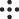, *p* < .05 women (CON to SSC).

There were significant LSR differences among conditions (p < .001; CON = 182.2 μL/min·dm^2^, IC95% 168.5 to 195.9 μL/min·dm^2^, SSA = 142.1 μL/min·dm^2^, IC95% 128.4 to 155.8 μL/min·dm^2^, SSB 158.1 μL/min·dm^2^, IC95% 144.4 to 171.8 μL/min·dm^2^, SSC = 159.0 μL/min·dm^2^, IC95% 145.3 to 172.7 μL/min·dm^2^). Additional analyses were performed incorporating sex as a variable to evaluate possible joint effects. Local sweating rate showed a significant interaction (p = .027) between sex and condition (Figure 3).

The means contrast analysis for the interaction showed that men in the CON condition presented a significantly higher local sweating rate compared to SSA (difference of 34.3 μL/min·dm^2^, *p* = .004), but no significant differences compared to SSB (difference of 4.5 μL/min·dm^2^, *p* = .693) or SSC (difference of 0.7 μL/min·dm^2^, *p* = .954). On the other hand, statistically significant differences were found between SSA and SSC conditions (difference of 33.6 μL/min·dm^2^, *p* = .005) and between SSA and SSB (difference of 29.8 μL/min·dm^2^, *p* = .012), while the SSB and SSC comparison evidenced no significant differences (difference of 3.9 μL/min·dm^2^, *p* = .735).

Significant differences between conditions were more evident in women; specifically, the CON condition presented a significantly higher local sweating rate compared to SSA (difference of 45.9 μL/min·dm^2^, *p* < .001), SSB (43.8 μL/min·dm^2^, *p* < .001) and SSC (45.8 μL/min·dm^2^, *p* < .001). On the other hand, no statistically significant differences were found between SSA and SSB (2.2 μL/min·dm^2^, *p* = .859), SSA and SSC (0.2 μL/min·dm^2^, *p* = .988), or between SSB and SSC (2.0 μL/min·dm^2^, *p* = .871) conditions.

## Discussion

The most salient finding of this research, which answers the primary objective of comparing the effect of three sunscreens on the rate of local sweating during exercise in heat, showed that the use of SSA with SPF 50 and a combination of both ultra violet A and B chemical sunscreen agents significantly decreases LSR (Figure 3). Specifically, this sunscreen reduces local sweat production compared to the control condition (no sunscreen). The results also revealed sex-specific differences, with women exhibiting greater decreases (*p* < .001) in LSR with the various sunscreen formulations, suggesting a potential sex-based variation in the sweating response to topical sunscreen application. Regarding the similar LSR between sexes in the control condition, contrary to typical findings, this unexpected result may stem from factors like body composition, fitness level, and acclimatization of our study population, as well as the localized scapular measurement approach versus whole-body assessments.

The use of SSA reduced LSR by 22.01% compared to the CON. Furthermore, SSB and SSC also exhibited reductions of 13.25% and 12.73%, respectively, in LSR relative to the CON condition, when males and females are analyzed together. In other words, the three sunscreens evaluated decreased the production of local sweating during exercise in heat with respect to not using protection, with SSA having the greatest effect on reducing local sweating. These LSR reductions should be interpreted in the context of inherent individual variation in sweating. Our repeated-measures design allowed us to control for this variability by having each participant serve as their own control across conditions. Additionally, by collecting samples from both sides of the body, we could account for potential lateral effects on sweat responses [10,22]. The measurement error associated with the reported local sweat rate (LSR) values in this study is relatively low, with a standard error of the mean (SEM) of approximately 1.8 μL/min·dm2 and fairly narrow 95% confidence intervals. This precision in the measurements supports the differences observed between the control and sunscreen conditions.

Beyond the chemical ultraviolet protection agents (organic, inorganic or natural chemical derivatives), the observed effect appears to be due also to some complementary ingredients that can generate an occlusive effect on the stratum corneum [31]. Regarding the ingredients of SSA, which showed the greatest differences versus the control, the use of dimethicone [13,14], c10-30 alkyl acrylate copolymer/ethyl acrylate [15], glyceryl stearate [16], hydrogenated coco-glycerides [17] and tocopheryl acetate [32] was detected (see Appendix 1). These components, to varying extents, could exhibit a cutaneous occlusive function that contributes to the effect of SSA in reducing sweating.

The protective role of sunscreen against UV-induced inflammatory effects on skin blood flow is crucial for skin care and health maintenance. Studies such as those by Wolf et al. [33] and Berry et al. [34] highlight this importance. However, our results further demonstrate that sun protection may indeed have an effect on thermoregulation, specifically by reducing the rate of local (scapular) sweating. Whether this local effect translates into reduced whole-body sweat rates and impaired thermoregulation and performance warrants further study. Meanwhile, this underscores the practical importance of selecting sun protection not only considering the prevention of skin damage but also the avoidance of occlusive ingredients than can moderate physiological responses.

Previous studies [10] had already evidenced that the use of a particular sunscreen had characteristics similar to those of an antiperspirant, reducing the rate of local sweating. This effect was corroborated in the present investigation. However, unlike the study by Aburto-Corona & Aragon-Vargas, in this case it is posited that rather than the chemical ultraviolet sunscreen agents (e.g. titanium dioxide, ethylhexyl methoxycinnamate, homosalate), what could be generating the “antiperspirant” effect are the complementary ingredients of the sunscreen that exhibit occlusive properties upon contact with the skin. This perspective was not addressed in previous studies [18,19]. The present study suggests a potential role of secondary or complementary ingredients in sunscreens with the observed inhibitory effects on sweating.

The main difference between the sunscreens is that SSA contains dimethicone, while SSB and SSC do not. Dimethicone is a type of silicone that can exhibit an occlusive effect on the stratum corneum [14,35,36]. Although SSB includes bis-peg-8 dimethicone, a different formulation, only SSA contains unaltered dimethicone. Additionally, SSA incorporates supplementary components including hydrogenated coco-glycerides and alkyl acrylate copolymers which may confer occlusive properties [15,17,32].

Consequently, it may be suggested that the amalgamation of dimethicone and auxiliary occlusive agents that differentiates the SSA formula is responsible for its superior occlusive properties. Despite comprising bis-peg-8 dimethicone, SSB does not incorporate unadulterated dimethicone or the other occlusive constituents present in SSA. Therefore, the unique combination of dimethicone and additional occlusive ingredients exclusive to SSA may be enabling it to form a more effective barrier on skin and decrease localized sweating during physical activity compared to SSB and SSC. Overall, the distinctive formulation of SSA allows it to stand out as having exceptional dermal occlusion abilities unmatched by SSB and SSC.

Sex differences in sweating have been extensively studied, with factors like hormones, body composition, skin surface area, sweat gland density/distribution, and cutaneous blood flow potentially playing a role [3]. While no significant differences were found between men and women in our control condition (*p* > .05), Aburto-Corona & Aragón-Vargas [10] reported higher localized sweating rates (LSR) in men (115.6 μL/min·dm2) compared to women (97.0 μL/min·dm2; *p* < .001). Their study also observed sex differences in LSR with organic sunscreen use, aligning with our findings. Oliveira [37] further demonstrated significant differences (*p* < .05) in total and local sweat rates across three regions between sexes. Contrasting with Ou-Yang et al. [18] and House & Breed [12] is not possible, as they evaluated only women or did not compare by sex, respectively.

This study presents the particularity that the LSR between men and women in the CON condition does not show significant differences. However, when compared to the other conditions (SSA, SSB and SSC), it is possible to detect that woman exhibit a greater effect in terms of LSR, being significantly lower. This difference observed between men and women when using sunscreen could be due to factors such as skin pH, if skins with higher acidity pH may facilitate the penetration of chemical agents [38] such as those present in sunscreens. This penetration of the product (sunscreen) could result in an obstruction of the sweat pore channels and thus affect sweat production. There are various positions related to skin pH, mentioning that men have lower acidity than women [39,40] or, on the contrary, that women have less acidic skin than men [41].

Additionally, another physiological difference that could explain the effect of using sunscreen in men and women could be due to the fact that women have a smaller skin thickness (epidermis and dermis) due to hormonal differences, lower muscle mass, and different dermal collagen distribution [42–44], facilitating the absorption of chemical agents in sunscreens that impact the LSR observed in this study.

In summary, this study shows reduced localized sweat rates with certain sunscreens, possibly due to occlusive ingredients like silicones. The greater reduction in women may stem from physiological sex differences. While sunscreen is essential during activity, formulations with high occlusive agent concentrations should be avoided. Future whole-body sweat rate assessments with full-body sunscreen application are warranted to fully characterize overall thermoregulatory impacts.

The potential impact of UV exposure on the observed results is an interesting consideration. While our study did not involve UV radiation, previous work suggests UV can influence sweating responses, likely through skin thermoregulatory and inflammatory mechanisms [33,45,46]. Incorporating UV may modulate the sweating rates we observed, either independently or via interactions with the sunscreen formulations. However, our goal was to isolate the direct effects of sunscreen compositions under controlled hot conditions. Including UV radiation was beyond this study’s scope but merits investigation in future research examining real-world sunscreen use scenarios with combined heat and UV stress.

A primary limitation of this study is the 20-min duration of the exercise protocol, which may not allow participants to reach a steady state in sweat rate. Although a longer duration could provide more stable measurements, the 20-min protocol was chosen based on previously validated studies and practical constraints [10,22]. Despite this limitation, the study design is robust and well-suited to our primary objective of evaluating the immediate effects of sunscreens on sweat rate.

Another limitation of this study is that we did not measure the presence of sunscreen components in sweat under different conditions or the concentration of sweat across trials. We acknowledge that understanding the presence and concentration of sunscreen components in sweat could provide additional insights; future research should include an analysis of sweat composition to determine if sunscreen components are present and how their concentrations vary across different conditions. This would help to elucidate the interactions between sunscreen formulations and the body’s sweat response.

Core body temperature was not measured in this study. Additionally, monitoring skin temperature at the measurement site would have provided valuable insight into the relationship between localized skin thermal dynamics and sweating responses under the different sunscreen conditions. Finally, the radiation was not considered. However, these limitations are mitigated by the study’s strong methodology and justification, which underscore the importance of examining the impacts of sunscreens on the sweating response as a key element of thermoregulatory function during exercise.

## Conclusion

The use of an organic sunscreen with SPF 50 during exercise in heat significantly reduces the rate of local sweating compared to not using sunscreen, with a greater reduction observed in women than in men. The inhibitory effect on sweating seems to be mainly due to some complementary ingredients with occlusive properties such as dimethicone, rather than UV filters. While the use of sunscreen is essential during outdoor physical activity, consideration should be given to avoiding sunscreens containing high concentrations of these occlusive ingredients to minimize potential interference with sweating thermoregulation, particularly during acute exercise bouts. Further studies measuring the effect on whole-body sweating rate over prolonged exercise durations are needed to quantify the full impact of sunscreens on thermoregulatory capacity during extended exercise in heat.

## Abbreviations

BW: Body weight
CON: Control
LSR: Local sweat rates
SD: Standard deviation
SPF: Sun protection factor
SSA: Sunscreen A
SSB: Sunscreen B
SSC: Sunscreen C
Tt: Tympanic temperature
USG: Urine specific gravity
UV: Ultraviolet

## Figures and Tables

**Table A1.** Characteristics of sunscreens (according to manufacturers).

	Sunscreen A (SSA)	Sunscreen B (SSB)	Sunscreen C (SSC)
Sun protection factor (SPF)	50	50+	50+
UV protection chemical agent	Homosalate (organic) – UVB filter Octocrylene (organic) – UVB filter Butyl methoxydibenzoylmethane (organic) – UVA filter Ethylhexyl salicylate (organic) – UVB filter Bis-ethylhexyloxyphenol methoxyphenyl triazine (organic) – broad spectrum filter (UVA/UVB) Phenylbenzimidazole sulfonic acid (organic) – UVB filter	Ethylhexyl methoxycinnamate (organic) – UVB filter Diethylamino hydroxybenzoyl hexyl benzoate (organic) – broad spectrum UVB filter Bistethylhexyloxyphenol methoxyphenyl triazina (organic) – UVA filter Methylene bis-benzotriazolyl tetramethylbutylphenol (organic) – broad spectrum UVB filter Isoamyl p-methoxycinnamate (organic) – UVB filter Titanium dioxide (inorganic) – broad spectrum UVB and UVA filter Octocrylene (organic) – UVB filter Butyloctyl salicylate (organic) – UVB filter	Bis-ethylhexyloxyphenol methoxyphenyl triazina (organic) – broad spectrum UVB and UVA filter Ethylhexyl salicylate (organic) – UVB filter Ethylhexyl methoxycinnamate (organic) – UVB filter Titanium dioxide (NANO) (inorganic) – broad spectrum UVB and UVA filter Butyl methoxydibenzoylmethane (organic) – UVA filter Ethylhexyl triazone (organic) – UVB filter
Other ingredients^†^	Water, Denatured alcohol, Glyceryl stearate, Hydrogenated coco-glycerides, Glycerin, Tocopheryl acetate, Tapioca starch, Cetyl alcohol, Phenoxyethanol, Dimethylsilyl silicate, Xanthan gum, Sodium stearoyl glutamate, Dimethicone, Methylparaben, Sodium hydroxide, C10-30 alkyl acrylate copolymer/ethyl acrylate, Trisodium EDTA, Sodium chloride, Fragrance.	Water, Dicaprylyl carbonate, Aluminum starch octenylsuccinate, Cetyl phosphate potassium, Glyceryl stearate, Coco-caprylate/caprate, Bis-peg-8 dimethicone, Cetyl alcohol, Diisopropyl sebacate, Sodium stearoyl glutamate, CI 77,492, Dimethicone vinyldimethicone crosspolymer, Isononyl isononanoate, Phenoxyethanol, Polymethylmethacrylate, Decyl glucoside, Fragrance (benzyl alcohol, benzyl benzoate, citronellol, geraniol, limonene), Silica, Caprylyl glycol, Isopropyl myristate, Acrylates/10-30 alkyl acrylate crosspolymer, Acrylate/styrene copolymer, Potassium hydroxide, Ethylhexylglycerin, Dimethyl silylated silica, Propylene glycol, CI 77,491, CI 77,499, BHT, Synthetic wax, Disodium EDTA, Oxothiazolidine, Perlite, Isopropyl titanium triisostearate, Pareth-12 c12-14, Glycol, Stearalkonium hectorite, Sodium hydroxide, Xanthan gum, Camellia sinensis leaf extract, Polyhydroxystearic acid, Propylene carbonate, Melanin, Pentylene glicol.	Water, Butylene glycol, Propylene glycol, Coco-caprylate/caprate, Polymethylmethacrylate, Cyclopentasiloxane, Bis-peg-18 methyl ether dimethyl silane, Acrylates/c10-30 alkyl acrylate crosspolymer, Alcohol, Arabidopsis thaliana extract, Ascorbyl palmitate, BHT, Caprylyl glycol, Carbomer, Citric acid, Decyl glucoside, Dimethicone, Disodium EDTA, Ergothioneine, Ethylhexylglycerin, Glycerin, Glyceryl oleate, Glyceryl stearate, Hexylene glycol, Hydrochloric acid, Lecithin, Micrococcus lysate, Fragrance, Phenoxyethanol, Phosphatidylcholine, Plankton extract, Polysilicone-11, Polysorbate 20, Potassium sorbate, Resveratrol dimethyl ether, Silica, Sodium chloride, Sodium cholate, Sodium hydroxide, Thermus thermophilus ferment, Tocopherol.

†, the order of the ingredients is from the highest to the lowest amount in the sunscreen.

## Data Availability

The data supporting the findings of this study are available in the Kérwá repository at [https://kerwa.ucr.ac.cr/handle/10669/91360]. The data can also be requested by contacting the corresponding author via e-mail at julian.garzon@ucr.ac.cr. The reuse of these data must contain a citation to this original article. There are no restrictions to data access.
